# An Interagency Collaboration to Facilitate Development of Filovirus Medical Countermeasures

**DOI:** 10.3390/v4102312

**Published:** 2012-10-19

**Authors:** Nicole Kilgore, Edwin O. Nuzum

**Affiliations:** 1 Chemical Biological Medical Systems Joint Vaccine Acquisition Program, 1564 Freedman Dr, Fort Detrick, MD 21702, USA; E-mail: nicole.r.kilgore.civ@mail.mil; 2 Office of Biodefense Research Affairs, DMID, NIAID, NIH, 6610 Rockledge, Bethesda, MD 20892, USA; E-mail: enuzum@niaid.nih.gov

**Keywords:** biodefense, Ebola, ebolavirus, FANG, *Filoviridae*, filovirus, Filovirus Animal Non-Clinical Group, Marburg virus, marburgvirus, medical countermeasures

## Abstract

The Filovirus Animal Non-Clinical Group (FANG) is a US interdepartmental and interagency group established to support and facilitate the advanced development of filovirus Medical Countermeasures (MCM), both vaccines and therapeutics. It is co-led by one representative from the Department of Defense (DoD), the first author, and one from the Department of Health and Human Services (HHS), the second author. The FANG membership includes operational level program staff and Subject Matter Experts (SME) from performing organizations as well as scientific staff and program managers from DoD and HHS funding and regulatory agencies. Focus areas include animal models, assays, reagents, product manufacture and characterization, and other interagency product development issues that will support Food and Drug Administration (FDA) licensure of safe and effective filovirus MCMs. The FANG continues to develop strategies to address broadly applicable and interagency product development challenges relevant to filovirus MCM development. This paper summarizes FANG structure and accomplishments and is meant to heighten community awareness of this government-led collaborative effort.

## 1. Introduction (and Background)

The Filovirus Animal Non-Clinical Group (FANG) is a US government interdepartmental and interagency group established to support and facilitate the advanced development of filovirus Medical Countermeasures (MCM). It was created to establish a forum for discussion, to develop as much consensus as possible, to minimize duplicate effort and to broaden awareness within the filovirus scientific and product development communities. The FANG is not a group that dictates actions regarding filovirus MCM research and development efforts. However, its deliberations, findings and recommendations should guide and impact US funding agency decisions regarding product development efforts. In support of the Research, Development, and Acquisition of Chemical, Biological, and Radiological Defense Materiel Memorandum of Understanding (CBR MOU) among the Department of National Defence of Canada, Defence of the United Kingdom of Great Britain and Northern Ireland, the Department of Defense of the United States, and the Department of Defence of Australia, the FANG includes participation from those countries with BSL-4 facilities. These integrated efforts are especially relevant to filovirus MCM development because Biosafety Level 4 (BSL4) facilities, though limited, are required for efficacy testing and reagent production.

The first interagency filovirus meeting was organized by Ms. Kathleen Berst in December 2008 when she was assigned to the Department of Defense (DOD) Chemical Biological Medical Systems -Joint Vaccine Acquisition Program (CBMS-JVAP). Initially, this group planned to meet quarterly. As the DoD and National Institutes of Allergy and Infectious Diseases (NIAID) Department of Health and Human Services (HHS) filovirus MCM initiatives advanced, more structure and more frequent meetings were needed as the number of efforts and complexity increased. The FANG was created and the group was chartered in July 2011. Currently, the FANG core and subgroup meetings occur at least monthly.

## 2. Results and Discussion

### 2.1. Purpose and Mission

As stated in the Charter, the FANG is meant to support the advanced development of filovirus Medical Countermeasures (MCM), both vaccines and therapeutics. High-level priorities are product development tools and other interagency product development (PD) issues relevant to FDA approval of filovirus MCMs. These efforts will result in strategies to address broadly applicable PD issues and to develop consensus recommendations that facilitate standardization of reagents, methods and procedures across multiple agencies and laboratories.

### 2.2. Goals

The FANG goals are to enhance communication, coordinate PD efforts and align PD regulatory and scientific resources regarding filovirus MCM development across multiple agencies; to form a cohesive interagency group composed of members at the “operational” level and appropriate SMEs to facilitate efficient decision making and implementation; to ensure there are no redundant PD efforts within and across agencies; to develop a unified US Government “message” that guides product sponsors and facilitates efficient product development; and to develop standardized protocols relevant to efforts such as, but not limited to, the following:

− Reagent production and characterization
− Assay and animal model development
− Assessments of challenge material, challenge dose, route of infection, *etc*.
− Measurement of critical endpoints such as viremia, pharmacokinetics, immune responses, correlates of protection, *etc*.
− Product manufacturing and characterization

### 2.3. Organization

Co-leads for the FANG and all subgroups represent both DoD and HHS. The principal government members include agencies from DoD [Joint Science and Technology Office (JSTO), CBMS-JVAP, United States Army Medical Research Institute of Infectious Diseases (USAMRIID), and Transformational Medical Technologies (TMT)], HHS [NIAID, Biomedical Advanced Research and Development Authority (BARDA), Centers of Disease Control and Prevention (CDC), and FDA (Center for Biologics Evaluation and Research (CBER), Center for Drug Evaluation and Research (CDER), and Office of Counterterrorism and Emerging Threats/Office of Chief Scientist (OCET/OCS)] and Department of Homeland Security National Biodefense Analysis and Countermeasures Center (NBACC). There are currently six subgroups that focus on specific technical areas critical to MCM development. They are: Animal Models, Challenge Material, Assays and Reagents, Therapeutic MCM PD, Vaccine MCM PD and Human Disease. The first three subgroups are active and productive. The other MCM groups will become active when candidate products are more advanced. The Human Disease group represents the greatest challenge in that one of its priorities is to obtain clinical data from filovirus disease outbreaks in humans that will help guide animal model development.

As each sub group developed and formulated their agenda, they have become critical components of the FANG for many reasons. In addition to the U.S. Government agencies listed above, partners of the CBR MOU and industry SMEs participate in subgroup meetings and discussions so their expertise can be utilized and to ensure that BSL4 labs are well represented. Discussions occur at a level of detail that would not be possible without the specific expertise of participating members. Problems and gaps are identified and plans and protocols are discussed to address them. Participation of members from BSL4 facilities is absolutely essential not only because of their expertise, but because they represent a critical and limited resource.

### 2.4. Accomplishments

Accomplishments are both organizational and scientific. Organizational accomplishments include finalization of the Charter, formation of the membership and subgroups and conduct of numerous core and subgroup meetings and three public presentations [1,2,3]. Scientifically, accomplishments include recommendations for filovirus challenge variants, documentation of the rationale for the intramuscular challenge dose used in efficacy studies, standardization of an Ebola virus (EBOV) plaque assay protocol, conduct of a non-human primate (NHP) study to evaluate virulence of two variants of Sudan virus (SUDV), identification of conditions and assays to grow and characterize challenge viruses and ongoing efforts to select the most appropriate animal species for relevant NHP efficacy models. Details of these accomplishments are not within the scope of this paper but will be published or otherwise made public at an appropriate time.

### 2.5. Future Direction

Future directions of the FANG and associated subgroups will be to continue ongoing efforts. Given the time required to make specific decisions regarding key questions and study objectives and then implement, complete and interpret those studies, attainment of definitive results for large efforts will be slow. Nonetheless, many concurrent efforts and progress towards definitive outcomes are ongoing. General areas of future endeavor include continued development, standardization and transfer of plaque assays and other assays for challenge material characterization; development of consistent and transferable challenge stock virus protocols; identification of positive control test materials; animal model refinement to include identification of relevant pathogenesis and efficacy study endpoints; and development of Good Laboratory Practices (GLP) and BSL4 capability.

## 3. Conclusions

The FANG has been successful in conducting constructive discussions, forming general consensus and making recommendations regarding current and future resource allocation that will support filovirus MCM development. Findings and recommendations developed by the FANG are being implemented by the filovirus community receiving filovirus MCM PD funding. Dissemination to the wider scientific community will occur in an appropriate manner as results and definitive decisions are obtained. Efforts, to ensure there are no duplicated efforts, are proceeding. Even though filovirus MCM candidates are relatively “immature”, and not beyond Phase 1 clinical testing, development of qualified and well-characterized resources that facilitate product development are costly and time consuming. Therefore, establishment of this integrated interagency endeavor has been invaluable to filovirus MCM advanced development efforts. For example, FDA participation in an informal scientific capacity provides a two-way dialogue beneficial to both FDA and non-FDA members. The approach and structure established by the FANG may be a viable approach for other government biodefense and emerging disease PD efforts.

Recent publications [4,5] evaluate and summarize the government’s success in vaccine development. Kendall Hoyt provides a historical assessment of innovation and PD success before, during and after World War II. She offers reasons why these efforts peaked during and immediately after the war and why success has decreased since the war. Common themes considered important include: transfer of “sticky knowledge”, focus, collaboration, coordination, integration and robust communities of practice. For example, sticky knowledge is knowledge that does not easily transfer from one facility to another. Absent personal relationships and mechanisms to transfer technical knowledge and successful practices between scientific communities and facilities, productive collaborations and synergistic outcomes are difficult at best. The FANG provides a structure and forum to address many of these issues as resource and product development efforts proceed.
